# Interpretable Artificial Intelligence for Analysing Changes in Gases in the Uterine Environment of Cows According to Physiological Structures in the Ovary

**DOI:** 10.1002/vms3.70252

**Published:** 2025-02-19

**Authors:** Ali Risvanli, Burak Tanyeri, Güngör Yildirim, Yetkin Tatar, Mehmet Gedikpinar, Hakan Kalender, Tarik Safak, Burak Yuksel, Burcu Karagulle, Oznur Yilmaz, Cebrail Barut, Mehmet Akif Kilinc

**Affiliations:** ^1^ Faculty of Veterinary Medicine, Department of Obstetrics and Gynecology Kyrgyz‐Turkish Manas University Bishkek Kyrgyzstan; ^2^ Faculty of Veterinary Medicine, Department of Obstetrics and Gynecology University of Firat Elazig Turkiye; ^3^ Department of Airframe & Powerplant Maintenance Firat University, Civil Aviation School Elazig Turkiye; ^4^ Faculty of Engineer, Department of Computer Engineer Firat University Elazig Turkiye; ^5^ Faculty of Technology, Department of Electrical Engineer Firat University Elazig Turkiye; ^6^ Faculty of Veterinary Medicine, Department of Microbiology University of Firat Elazig Turkey; ^7^ Faculty of Veterinary Medicine, Department of Obstetrics and Gynecology University of Kastamonu Kastamonu Turkiye; ^8^ Faculty of Veterinary Medicine, Department of Obstetrics and Gynecology University of Siirt Siirt Turkiye; ^9^ Faculty of Veterinary Medicine, Department of Obstetrics and Gynecology University of Bingol Bingol Turkiye

**Keywords:** corpus luteum, cow, follicle, gas, Metrisör

## Abstract

The objective of the present study was to examine the relationship between the gases in a cow's uterine environment and its ovarian physiological structures using the sunflower optimisation algorithm (SFOA) deployed in a device called Metrisör, developed by our project team. A total of 500 uteruses obtained from slaughtered cows served as the experimental sample. Gas measurements were taken from 489 uteruses with no clinical metritis or microbiological growth. Additionally, the diameters of the corpus luteum and follicles in the ovaries were measured using callipers. These results were then analysed based on the presence or absence of a corpus luteum (CL) and follicles larger or smaller than 1.5 cm. According to uterine gas fluctuations, the presence and absence of CL could be detected at rates of 80.60% and 79.60%, respectively. Also, based on uterine gas changes, the presence of ovarian follicles larger than 1.5 cm was determined 82% of the time, and the presence of follicles smaller than 1.5 cm was determined 80% of the time. In conclusion, it was found that different stages of a cow's sexual cycle might involve changes in uterine gases. Thus, the data from this study may enable the development of a new estrus detection method for cows.

## Graphical abstract

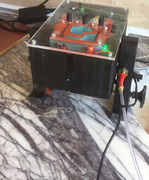

## 1 Introduction

Previously, it was believed that the uterus remained sterile during pregnancy and that it became contaminated with nonspecific bacteria from animals and the environment postpartum. Current evidence, however, suggests that the uterus is not sterile and houses specific microorganisms, or commensal bacteria, that adapt to the endometrium. Still, compared to the microbiota of the intestine or vagina, the uterine microbiota is less abundant in terms of microbial composition (Chen et al. 2017; Baker, Chase, and Herbst‐Kralovetz 2018; Moreno and Franasiak 2017; Pelzer et al. 2017).

Microorganisms produce metabolites such as amino acids, proteins, carbohydrates, vitamins, acetone, ethanol, organic acids, antibiotics, toxins and alkaloids (Onbasılı and Aslım 2011). During their growth, these organisms can generate volatile organic compounds that are specific to their species (Amann, Spanel, and Smith 2007). Gases released during the bacterial growth cultured in an airtight bioreactor were collected and analysed through gas chromatography, demonstrating that they release certain trace gases (Bunge et al. 2008; Moularat et al. 2008; Zhu et al. 2010; Lechner et al. 2005; Shin et al. 2009).

The use of electronic nasal devices, which detect volatile sulphur compounds (such as hydrogen sulphide, dimethyl sulphide, methyl mercaptan and sulphur dioxide) produced by oral bacteria that cause bad breath in humans, is becoming more common. These compounds are typically detected using the gas chromatography technique (Aylıkçı and Çolak 2013; Kini et al. 2012).

Gas sensors can be developed from a range of materials, including graphene, carbon nanotubes, conducting polymers, and metal oxides (Na et al. 2016; Kokabu, Inoue, and Matsumura 2015; Alizadeh et al. 2015; Kim and Lee 2014). Conducting polymer gas sensors, relying on conductivity, continues to hold validity in terms of sensitivity. However, their repeatability is hampered due to moisture susceptibility (Alizadeh et al. 2015). Metal‐oxide semiconductor (MOS) gas sensors, another type of sensor, prove to be the most suitable for industrial use, owing to their low cost and mature technologies, despite their sensitivity performance not being as commendable as others (Kim and Lee 2014). Furthermore, in recent years, Micro‐Electro‐Mechanical System (MEMS) sensors have been gaining rapid traction, especially for their small size, high sensitivity, and low power consumption (Khoshnoud and de Silva 2012).

Our team developed a device called Metrisör, which is noted for its ability to accurately detect metritis in the uterus, bacterial growth in the uterus, and bacterial growth in uteruses exhibiting metritis. This detection process involves sensing gases emitted during microorganism growth utilising sensor technology. Additionally, it can detect up to 80% of *Escherichia coli*, *Staphylococcus aureus*, *Coagulase‐negative staphylococcus*, *Trueperella pyogenes*, *Bacillus* spp, *Clostridium* spp, and *Fusobacterium necrophorum* bacteria known to cause metritis (Risvanli et al. 2024).

In cow breeding, the goal is to produce one calf per year. To achieve this, effective estrus monitoring is crucial. Consequently, understanding the signs of estrus and accurately detecting them is important (Youngquist and Threlfall 2007). Modern animal husbandry has made the detection of estrus in cows and heifers both more difficult and more critical. Yield losses are often due to unsuccessful estrus detection. A variety of methods are employed for this purpose, leading to extensive efforts to accurately determine the estrus of cows and heifers and to inseminate and conceive them in a timely manner. The most frequently used methods include observation, pedometers, devices for detecting mounting activity, the use of a teaser animal, cameras, heat meters, resistance metres, hormone analyses, and others (Noakes, Parkinson, and England 2009; Roelofs et al. 2010). However, recently, there's been a growing interest in techniques using electronics and various sensor technologies.

This study aims to examine the relationship between gases in the uterine environment of cows and the physiological structures on the ovary using the Metrisör device. This device operates using the sunflower optimisation algorithm (SFOA) and sensor technology. By analysing the gas changes relative to the sexual cycle period of the cows, we intend to determine if a new estrus detection method can be developed for cows.

## 2 Materials and Methods

The study utilised 500 uteruses gathered from cows slaughtered at three different slaughterhouses in xxx due to infertility. The cows varied in age, breed and lactation periods. To ensure a sterile procedure, swabs were taken from the uteruses for microbiological tests and transferred to a microbiology laboratory via a cold chain transport medium. During the collection of swab samples, asepsis and antisepsis standards were strictly observed. Subsequently, gas measurements were taken from 489 uteruses–all without clinical metritis and with no microbiological growth–using a device known as a Metrisör. Furthermore, the diameters of the corpus luteum (CL) and follicles in the ovaries were measured with callipers. In instances where more than one CL and follicle were detected, the diameters of the largest CL and follicle were utilised for the measurement base.

### 2.1 Isolation and Identification of Bacteria

Bacterial isolation and identification were carried out in the microbiology laboratory of Fırat University, Faculty of Veterinary Medicine. Uterine swab samples, collected under sterile conditions into transport medium tubes (Cary Blair Medium, LAB505, Lab M), were delivered to the microbiology laboratory under a cold chain. These samples were inoculated onto 5% blood agar (Oxoid) and MacConkey agar (Oxoid). The culture media were incubated under aerobic conditions and 5% CO_2_ at 37°C for 48 h. For isolating anaerobic bacteria, swab samples were inoculated onto Cooked Meat Medium (Oxoid) and incubated at 37°C for 72 h. The anaerobic environment was maintained using anaerobic gas kits (Anaerocult_A Merck 1.13829 CE) and an anaerobic jar (Merck 1.16387.0001 CE). To test whether an anaerobic environment was provided, Anaerocult test sticks (Merck 1.15112.0001 CE) capped with a blue‐coloured tip were placed into the anaerocult jars. Morphological characteristics of colonies, Gram stain reactions, oxidase, catalase, haemolysis, and other biochemical properties of the isolated microorganisms were examined and identified. Furthermore, a PCR test was conducted to confirm the identification of *Fusobacterium necrophorum* (Werner et al. 2012; Ozgen et al. 2015).

### 2.2 Detection of Clinical Metritis

The presence or absence of clinical metritis in the uterus was determined, as described by Senünver and Nak (2012). Accordingly, uteruses with symptoms such as cervical discharge, asymmetry between the uterine horns, thickened wall, edematous changes, discolouration, and increased tone and volume were defined as having clinical metritis.

### 2.3 Measurement of Intrauterine Gases

The device named Metrisör (Turkish Patent No: TR 2022 012613 B, PCT/TR2023/050683), developed by the project team presented in this study, was used to measure intrauterine gases. The sensors are metal‐oxide and electrochemical types, and it is sensitive to gases such as CO_2_, NO_2_, NH_3_, benzene, ethanol, CH_4_, H_2_, CO, NH_4_, toluene, formaldehyde, O_2_, C_4_H_10_, acetone, N‐hexene, methanol, and general volatile organic compound. The device also contains a digital sensor that measures temperature and humidity within the gas measurement environment. The probe of the Metrisör, operating under these conditions, was inserted into the uteri of live animals obtained from a slaughterhouse. Gas was then drawn from the uterus into the device's chamber by activating the vacuum arm. All sensors were exposed to the gas drawn from the uterus, facilitated by the device's circulation arm. For each uterus, 20 measurements were taken, ensuring the temperature within the device remained around 33–35°C. If this temperature was exceeded, the measurements were halted, and the device was allowed to cool. After each measurement, the device's discharge lever was pulled to expel the gases from the chamber, which was then replenished with fresh air.

After the completion of gas measurements, samples for microbiological tests were taken from the uteruses. The swabs used for these samples were sterile and were sent to the microbiology laboratory in transport media. The tests that were performed aimed to uncover the correlation between gas measurements obtained from uteruses with clinical metritis, the absence of microbiological growth, and the physiological structures on the ovary; these tests are described below.

### 2.4 Sunflower Optimisation Algorithm (SFOA)

The SFOA is a population‐based algorithm inspired by nature, mimicking the sun orientation movements of sunflower plants. Its fundamental principle is attaining the optimal solution, referred to as the ‘sun’. SFOA has been applied to many problems, with results revealing its success. Qais, Hasanien, and Alghuwainem (2019) used SFOA, obtaining remarkable results in the modelling and simulation of photovoltaic modules using varying parameters. In 2020, Yuan et al. suggested the SFOA method for optimal parameter selection of Proton Exchange Membrane Fuel Cell (PEMFC) models. Performance analysis was performed on two practical models, the NedSstack PS6 and Horizon 500‐W PEMFCs, with results compared to experimental data and state‐of‐the‐art methods like the Seagull Optimisation Algorithm (SOA), Multiverse Optimisation (MVO), and Mixed Frog Leap Algorithm (SFLA). SFOA reportedly yielded superior results. Raslan et al. (2021) proposed an improved SFOA model for the best cluster head selection in wireless sensor networks. The results suggested better performance in energy consumption and network lifespan compared to other methods. Emami (2022) proposed an enhanced SFOA model for task scheduling in cloud systems, with improved outcomes for make span and energy consumption. Barut et al. (2024) recommended a rule‐based method for the task scheduling problem in cloud systems. They used SFOA to derive various rules concerning task groups and available virtual machines in the cloud environment and found it successful in rule extraction. Ulucan et al. (2023) developed an explainable model for predicting mixed form and compressive strength classes of concrete with recycled aggregates, comparing it favourably to state‐of‐the‐art methods.

The SFOA is based on the behaviour of sunflowers as they follow the sun's rays (Gomes, da Cunha, and Ancelotti 2019). Here, sunflowers symbolise candidates aiming to find the best position by heading towards the sun. In SFOA, candidates distant from the sun attempt to draw closer quickly using larger orientations, while candidates near the sun strive to maintain their existing position.

As they update their positions, they can generate new candidates by pollinating with neighbouring individuals. Although millions of pollinations occur in nature, the algorithm simplifies this by assuming each sunflower performs one pollination.

In SFOA, the process starts by creating an initial population. The individual nearest to the solution in this initial population is designated as the sun. Once the sun is identified, neighbouring individuals pollinate among themselves in relation to the position of the sun to form new individuals. This production of new individuals increases the search space size, and to counter this, sunflower individuals are regularly eliminated. The updating and generation of new individuals continue iteratively until a termination criterion is attained.

One essential principle in the algorithm is the depiction of sunflower orientation toward the sun. The sun (C*), representing the immediate best solution within the population, serves as a reference for the other sunflower individuals (*C_i_*). The sun orientation of a population containing *N* individuals is denoted by *R_i_*, and its general expression is presented in Equation 1. Within the algorithm, individuals’ orientation towards the best solution is expressed by Equation 2. This orientation relies on two fundamental parameters: the inertia coefficient *φ* and *P_i_*, which signifies the probability of pollination between the *i*th and the (*i*–1)th individuals. As inferred from the general expression, individuals closer to the sun tend to orient towards the sun with smaller steps, while those farther away attempt to orient with larger steps.

(1) $$\;R_{i}=\frac{C^{*}-C_{i}}{\left|\left|C^{*}-C_{i}\right|\right|},\;i=1,2,\cdots ,N,$$

(2) $$\;S_{i}=\varphi P_{i}\left(∥P_{i}+P_{i-1}∥\right)∥P_{i}+P_{i-1}∥.$$

In SFOA, each iteration involves the removal of some individuals that are distant from the sun, based on the removal rate (O), with new individuals generated in their place. The entry of different individuals into the population at every iteration aids in discovering novel points. Beyond this, when determining the new positions of other individuals, it is vital to consider the maximum orientation amount value so as not to disregard global points. This fundamental principle is facilitated by Equation 3. In this equation, *X*_max_ and *X*_min_ refer to the upper and lower bounds, whereas *S*_pop_ represents the total population of individuals. The creation of the new individual is determined by the computation outlined in Equation 4.

(3) $$\;d_{\max}=\frac{\left|\left|C_{\max}-\;C_{\min}\right|\right|}{2S_{\mathrm{pop}}},\;$$

(4) $$\;{\vec{C}}_{i+1}={\vec{C}}_{i}+S_{i}{\vec{R}}_{i}.$$

### 2.5 The SFOA‐Based Rule Mining System

Certain methods used for classification are often described as black boxes because it is impossible to ascertain a clear relationship between the result and the data. Even though the outcome is accurate, the influencing elements remain unclear. As the quantity of parameters in a data set containing numerical information escalates, defining relationships between them becomes progressively challenging. In rule mining, both the factors that impact the obtained result and their values are discernible. Therefore, through the rules derived, one can identify which parameters in the data set influence the result.

Metaheuristics usually involve randomisation, which causes the results to vary. In this study, a rule‐based Parallel SFOA algorithm is proposed to minimise this problem. With the parallel method, two SFOA processes are created in the search space, and these processes run independently of each other. SFOA is based on a different representation for each solution in the population of *n* individuals. In this representation, a solution *X_i_* of dimension *d* (number of attributes), $y_{i}^{t},\;y_{i}^{s}\;\mathrm{ve}\;y_{i}^{k}$ (i = 1, 2, ⋯, *n*) contains three subdata. $y_{i}^{t}=\left(y_{1}^{t},y_{2}^{t},\;\cdots ,y_{j}^{t},\cdots ,y_{d}^{t}\right)$ holds binary data for each attribute of a solution. Whether a *j*th attribute is to be added to the rule of a solution $y_{j}^{b}$ is determined by its value. A random generator function in the initial population $y_{j}^{t}=\mathrm{rand}\left(0,1\right)$, and a predefined threshold value ‘*δ*’. This process is calculated by Equation 5.

(5) $$\;y_{j}=\left\{\begin{array}{c} 1\;\left(\mathrm{if}\;y_{j}\;\mathrm{used}\;\mathrm{in}\;\mathrm{the}\;\mathrm{rule}\right)\;then\;y_{j}^{t}>\;\delta ,\;\delta \;\varepsilon \;\left[0,1\right]\;\\ \;\\ 0\;\left(\mathrm{if}\;y_{j}\;\mathrm{not}\;\mathrm{used}\;\mathrm{in}\;\mathrm{the}\;\mathrm{rule}\right)\;then\; \end{array},\right.\;$$

$\;y_{i}^{a}=\left(y_{1}^{a},y_{2}^{a},..y_{j}^{a},..x_{d}^{a}\right)$ denotes the lower limits calculated for each attribute of the rule of a solution; $y_{i}^{u}=\left(y_{1}^{h},y_{2}^{h},..y,..y_{d}^{h}\right)$ indicates the upper limits of the attributes in the corresponding solution rule ($y_{d}^{a},\;y_{d}^{h}\;\varepsilon \;R$). Subvalue in the search space $N^{b}$ and its upper‐value $N^{h}$ for attribute ‘*j*’, which is ‘$N^{b}\leq y_{j}^{h}\leq N^{h}$’ condition is always valid in each iteration. During the iterations, each candidate has its $y_{i}^{t},\;y_{i}^{b}\;\mathrm{ve}\;y_{i}^{h}$ recalculate the values. At the end of the iterations $x_{j}^{b}>\;\delta$ every qualification that fulfils the condition ‘$X_{i}$’ is added to the rule of the solution. Rule mining optimisation is performed separately for each class in the dataset. This means that the main data set (search space) is divided into subspaces for each class, and each optimisation process takes place in the corresponding subspace. Optimisation of a solution $y_{i}^{t},\;y_{i}^{a},\;\mathrm{ve}\;y_{i}^{h}$ values is checked for each data in the relevant search space and used to calculate the fitness value of the objective function. For example, in a rule mining optimisation process for class ‘S’, suppose that a solution uses attributes 2, 3, and 4 in its rule $\;(y_{2}^{t},\;y_{3}^{t},\;\mathrm{ve}\;y_{4}^{t}>\;\delta ,\;\mathrm{in}\;i.\;\mathrm{iteration}).$ In this case, the ‘*k*’ data of the candidate solution ($T_{k_{1}},\;T_{k_{2}},\;\cdots ,\;T_{k_{m}}$), will be checked according to Equation 6.

(6) $$\begin{array}{ccc} & & \mathrm{if}\;y_{1}^{a}\leq D_{k_{1}}\leq \;y_{1}^{h}\;\mathrm{and}\;y_{2}^{a}\leq \;D_{k_{2}}\leq \;y_{2}^{h}\;\\ & & \;\mathrm{and}\;y_{3}^{a}\leq \;D_{k_{3}}\leq \;y_{3}^{h}\;\mathrm{then}\;S. \end{array}$$

This calculation process is used to calculate the fitness value of the objective. In this study, the ‘accuracy’ value of the classification is taken as the objective function $\;y_{i}.$ In order to find the ‘accuracy’ value of the solution, the condition in Equation 6 is checked one by one for each data belonging to the relevant ‘*S*’ class. As a result of these operations, true‐positive (CP), true‐negative (DN), false‐positive (FP) and false‐negative (FN) values are obtained. For this purpose, as shown in Table 1, the ‘if’ part (before ISE) and the ‘if’ part (after ISE) of the relevant rule are evaluated separately. Then, the ‘accuracy’ value used as the objective function is calculated as in Equation 7 based on the DP, DN, YP, and YN values of the relevant rule.

(7) $$\mathrm{Accuracy}=\frac{TP+TN}{TP+TN+FP+FN}.\;$$

**TABLE 1:** Calculation of TP, TN, FP and FN values.

‘IF’	‘THEN’	Process
True	True	Increase by 1 TP
True	False	Increase by 1 TN
That's right	False	Increase by 1 FP
False	True	Increase by 1 FN

*TP* is the number of cases where both the left and right sides of the rule are true, *FN* is the number of instances where both sides of the rule are false, *FP* represents the number of cases where the left side of the rule is true, and the right side is false, and *FN* denotes the instances where the left side of the rule is false, and the right is true. The pseudo‐code depicted in Algorithm 1 visualises the operation of the processes mentioned above, expanding on the rule extraction‐based SFOA. The algorithm operates in two key stages. First is the training phase, where the SFOA is adapted and executed to the fitness value for the training data sets. At the end of the algorithm iterations, the rules that were obtained are then retested for the test data. This same evaluation procedure is performed once again for the test data (Table 2).

**TABLE 2:** Algorithm 1: **AOA pseudocode**.

Get Dataset and normalise it Get iteration number, Population size, upper, lower base and elimination Rate Create the initial population while(*i* <iteration number CalculateFitness (Population) S← FindSun(P opulation) OrientationtoSun () for *j = 1*' to population size Pollination() CalculateOrientationOfPlant() EleminateSunFlowers(Elimination_Rate,*i*) EvaluateNewSunflowers (S) end for End while *Try Rules For Tes tData()* 16. ShowBestRules()

## 3 Results

Experiments were conducted separately for CL presence and follicle size. The CL dataset comprised 489 data points, with 125 labelled as ‘A’ (Absence) and 364 labelled as ‘P’ (Presence). This dataset contained a total of 19 different parameters. Meanwhile, the follicle dataset comprised a total of 394 data points, of which 283 were labelled as ‘O’ (over 1.5 cm), and 111 were labelled as ‘L’ (less than 1.5 cm). This dataset also incorporated 19 distinct parameters. Representative samples of the datasets used are displayed in Tables 4 and 5.

In the experiments, the outcomes derived from the rule‐based Parallel SFOA method were compared with standard rule extraction approaches and classification methods. Standard rules extraction methods included JRIP, FURIA, OneR, ConjunctiveRule, DecisionTable and ZeroR, while Naive Bayes, Support Vector Machine, KStar, Vote, HyperPipes, J48 and RandomTree were employed as classification methods.

The rule‐based Parallel SFOA method was initially trained with the training data from the dataset, generating several candidate rules. Due to the possibility of extracting numerous rules in the experiments, a certain quantity of the best rules was chosen in each experiment and then tested in the subsequent stage. This study employed the accuracy metric to ascertain the effectiveness of a candidate. An initial evaluation of the test data over the established rules was conducted, and the accuracy value of each rule was determined. This accuracy value was then compared with other methods.

The trials ran on Python programming language on a computer equipped with an Intel(R) Core(TM) i5 processor and 8.00 GB of memory. The dataset was partitioned such that 20% was allocated for testing and 80% for training. The other parameters utilised in the rule‐based Parallel SFOA implementation are outlined in Table 3.

**TABLE 3:** Other parameters of rule‐based SFO and experiments.

Parameter	Value
Population size	30
Pollination rate	2.5%
Mortality	20%
Iteration	300
Independent experiment	5

**TABLE 4:** Follicle size data set.

MQ8	MQ3	NH3	T2602	NO2	T822	HCHO	T2620	MS1100	MQ7	MQ6	MQ4	MQ135	TEMP1	NEM1	Class
216	539.2	594.8	932.4	98.8	843	232.4	963.2	220.5	137	283.8	110.8	930	34.8	27.4	**A**
198.7	519.1	647.3	927.8	77.7	837.3	162	962.8	209	129	269.9	104.6	930	36	24.9	**A**
198.5	511.1	600	928.4	77.4	862	137.4	963	206.4	130.3	270	109.4	931.4	38	26	**U**
192.09	498.18	610.91	923.45	70.55	853.55	121.55	960	199.36	126.91	264.45	106	931.55	39	24.45	**U**
187.82	486.73	622.09	923.36	69.18	842.36	112.45	956	193.45	125.45	260.73	104.09	931.09	40	23	**A**
187.27	482.09	631.64	920.09	69.55	836.91	108.73	951.36	190	124.18	259.45	102	930.09	41	22.09	**U**
262.5	525.7	769.2	941.2	230.5	781.2	127.7	896.1	421.3	251	321.8	251	927	30	25.8	**U**
266.9	525.4	777	940	227.7	697	134.9	889	410.5	251	327.1	240.8	927.8	30.8	27.4	**U**
273.7	527.4	778.3	935.9	225.4	761.1	119.6	882.5	402.6	250.6	334.7	227.8	924.5	31	29	**A**
287	532.36	769.82	934.09	214.55	762.64	125.18	869.36	388.55	251.64	348.27	218.27	925.82	31.27	29	**A**
252.82	573.18	537.18	933.36	53	887	133.36	955.09	278.45	159.91	328.64	129.73	900	33	31	**A**
180.8	490.6	651.8	911.4	62.1	830	87.7	945.1	175	123.8	252.6	96.2	886.8	36	27.7	**U**
177.3	479.8	653.7	910.3	63	827.4	86	944.6	170	119.7	249.3	93.4	873.5	36.2	27	**A**
171.7	466.5	666	907.9	63.1	813.7	83.1	942	161.6	116.9	244.8	90.7	869.3	37	25.8	**A**
177.2	471.2	645	926.9	66.2	814	107.5	942.8	169.7	121.1	250.4	95	893.6	38	26.6	**A**
173.9	461.4	656.4	916.7	64	817	92.8	941.9	166.3	118.8	247.1	90.7	895.4	39	25	**U**
172.2	457.3	661.1	913.4	64	812.8	89.2	940.1	162.9	118	245.6	89.3	896	40	24.4	**A**
265.5	582.5	544.3	952.7	52.9	868.2	249.6	949.9	297.8	160	340.8	127.4	893.1	34	31.4	**A**
216	542.5	597.7	932.3	56.5	850.6	123.2	952.6	216.1	140	287.2	110	878	34	31.5	**A**

**TABLE 5:** CL presence data set.

MQ8	MQ3	NH3	T2602	NO2	T822	HCHO	T2620	MS1100	MQ7	MQ6	MQ4	MQ135	TEMP1	NEM1	Class
275.2	590.9	513.6	937.8	51.7	893.3	186.2	961	348.6	165.5	348.9	139.6	935.1	32	27.2	**Y**
216	539.2	594.8	932.4	98.8	843	232.4	963.2	220.5	137	283.8	110.8	930	34.8	27.4	**V**
198.7	519.1	647.3	927.8	77.7	837.3	162	962.8	209	129	269.9	104.6	930	36	24.9	**V**
198.5	511.1	600	928.4	77.4	862	137.4	963	206.4	130.3	270	109.4	931.4	38	26	**Y**
192.09	498.18	610.91	923.45	70.55	853.55	121.55	960	199.36	126.91	264.45	106	931.55	39	24.45	**V**
187.82	486.73	622.09	923.36	69.18	842.36	112.45	956	193.45	125.45	260.73	104.09	931.09	40	23	**V**
187.27	482.09	631.64	920.09	69.55	836.91	108.73	951.36	190	124.18	259.45	102	930.09	41	22.09	**V**
236.6	507.7	772.8	942.6	260.4	779.6	197.4	922.8	453.6	239.2	287.3	333.3	926.9	31	24	**Y**
262.5	525.7	769.2	941.2	230.5	781.2	127.7	896.1	421.3	251	321.8	251	927	30	25.8	**Y**
266.9	525.4	777	940	227.7	697	134.9	889	410.5	251	327.1	240.8	927.8	30.8	27.4	**Y**
273.7	527.4	778.3	935.9	225.4	761.1	119.6	882.5	402.6	250.6	334.7	227.8	924.5	31	29	**V**
282	531.4	771.6	935.7	218.2	771.3	124.9	875.6	394.7	252	343.7	224	925.6	31	29.2	**Y**
287	532.36	769.82	934.09	214.55	762.64	125.18	869.36	388.55	251.64	348.27	218.27	925.82	31.27	29	**V**
294.7	536.1	760.2	933.1	211.6	722.3	122	865.6	383.9	252	355.5	212.3	926.8	32	29.6	**V**
252.82	573.18	537.18	933.36	53	887	133.36	955.09	278.45	159.91	328.64	129.73	900	33	31	**V**
207.2	530.7	626.3	912.1	60.4	841.6	101.8	950	204.2	136.5	277.4	104.9	861.9	34	28.3	**Y**
192	510.1	631.3	910.6	61.7	842.9	90.3	947	184	130.5	262	102	864.9	35	29.2	**V**
180.8	490.6	651.8	911.4	62.1	830	87.7	945.1	175	123.8	252.6	96.2	886.8	36	27.7	**V**
177.3	479.8	653.7	910.3	63	827.4	86	944.6	170	119.7	249.3	93.4	873.5	36.2	27	**V**

The metric values from the experiments on the follicle dataset for the four best candidates are detailed in Table 6. The rules extracted from the successful candidates during the training phase are displayed in Table 7. When analysing these results simultaneously, class L demonstrates the highest accuracy value. Optimal values for class L are [0.785, 0.789, 0.984], while those for class O are [0.772, 0.00, 0.00]. The experiment results yielded more than one rule of identical values.

**TABLE 6:** The accuracy values of the best candidates (Training stage), according to follicle size.

Class	Accuracy
L	0.785
	0.785
	0.785
	0.785
O	0.772
	0.772
	0.772
	0.747

**O**: over 1.5 cm, **L**: less than 1.5 cm.

**TABLE 7:** The created rules for the candidates (Training stage), according to follicle size.

Class	CND	Rule	Accuracy
L	1	if((266 ≤ MQ3 ≤ 637) and (516 ≤ NH3 ≤ 847)) Then L	0.785
	2	if((265 ≤ MQ3 ≤ 637) and (515 ≤ NH3 ≤ 848)) Then L	0.785
	3	if((266 ≤ MQ3 ≤ 637) and (516 ≤ NH3 ≤ 847)) Then L	0.785
	4	if((265 ≤ MQ3 ≤ 637) and (515 ≤ NH3 ≤ 847)) Then L	0.785
O	1	if((246 ≤ MQ8 ≤318) and (559 ≤ NH3 ≤747) and (905 ≤ T2602 ≤972) and (57 ≤ NO_2_ ≤172) and (527 ≤ T822 ≤836) and (840 ≤ T2620 ≤920) and (302 ≤ MS1100 ≤394) and (200 ≤ MQ6 ≤344) and (31 ≤ TEMP1 ≤32)) Then O	0.722
	2	if((263 ≤ MQ8 ≤299) and (529 ≤ NH3 ≤781) and (902 ≤ T2602 ≤970) and (58 ≤ NO2 ≤98) and (543 ≤ T822 ≤744) and (213 ≤ HCHO ≤244) and (856 ≤ T2620 ≤907) and (331 ≤ MS1100 ≤333) and (225 ≤ MQ7 ≤225) and (770 ≤ MQ135 ≤901)) Then O	0.722
	3	if((57 ≤ NO2 ≤219) and (128 ≤ HCHO ≤222) and (736 ≤ T2620 ≤860) and (149 ≤ MS1100 ≤254) and (199 ≤ MQ7 ≤206) and (206 ≤ MQ6 ≤251) and (804 ≤ MQ135 ≤854) and (31 ≤ TEMP1 ≤39)) Then O	0.722
	4	if((183 ≤ MQ8 ≤206) and (875 ≤ T2620 ≤948) and (127 ≤ MQ7 ≤217)) Then O	0.747

**O**: over 1.5 cm, **L**: less than 1.5 cm.

The most successful rules obtained have been tested with the relevant data. Tables 8 and 9 showcase a comparison of the results derived from our proposed method against standard rule‐based and classification methods. To provide a more transparent evaluation, we used the weighted values of the obtained results. The data presented in Tables 8 and 9 indicate that the rule‐based Parallel SFOA method is decidedly superior to all other methods. Given that the rule‐based Parallel SFOA is a method based on random search, a statistical evaluation is required. Consequently, the statistical results from ten independent experiments with the rule‐based Parallel SFOA method are displayed in Table 10. Upon analysis of these statistical results, it becomes evident that the values of Class O surpass those of Class L.

**TABLE 8:** The comparative results of the rule‐based Parallel SFO and well‐known rule‐based methods (weighted), according to follicle size.

Method	Accuracy
Rule‐based Parallel SFO	78.83
Jrip	71.32
Ridor	71.07
Furia	69.04
OneR	65.99
ConjuctiveRule	71.83
DecisionTable	71.83
ZeroR	71.83

**TABLE 9:** The comparative results of the proposed rule‐based Parallel SFO and standard classification methods (weighted), according to follicle size.

Method	Accuracy
Rule‐based Parallel SFO	78.83
NB	**64.47**
SVM(SMO)71.8274	71.83
KStar	63.71
Vote	71.83
HyperPipes	71.32
J48	70.81
RandomTree	65.74

**TABLE 10:** The statistical accuracy results of all the rule‐based Parallel SFO experiments, according to follicle size.

	Class L	Class O
Best	80.00	82.00
Worst	27.00	63.00
Mean	68.00	73.00
Median	70.0	73.00
Std	0.1	0.04

**O**: over 1.5 cm, **L**: less than 1.5 cm.

The metric values for the top four candidates obtained from the experiments performed on the CL dataset are presented in Tables 11 and 12, which show the rules extracted from successful candidates. Upon evaluating these results, the highest accuracy value was observed for class A. The best values for class A are [0.796, 0.786, 0.776], and for class P, they are [0.786, 0.776, 0.765]. The experiment results yielded more than one rule with the same values.

**TABLE 11:** The accuracy, precision and recall values of the best candidates (Training stage), according to presence CL.

Class	Accuracy
A	0.796
	0.786
	0.765
	0.776
P	0.786
	0.776
	0.765
	0.755

**A**: Absence CL, **P**: Presence CL.

**TABLE 12:** The created rules for the candidates (Training stage), according to presence CL.

Class	CND	Rule	Accuracy
V	1	if((259 ≤ MQ3 ≤642) and (61 ≤ MS1100 ≤449) and (19 ≤ NEM1 ≤36)) Then P	0.796
	2	if((61 ≤ MS1100 ≤404) and (183 ≤ MQ6 ≤387) and (24 ≤ TEMP1 ≤41) and (19 ≤ NEM1 ≤36)) Then P	0.786
	3	if((739 ≤ T2620 ≤963)) Then P	0.785
	4	0.776, 0.776, 1.000, if((111 ≤ MQ8 ≤ 320) and (259 ≤ MQ3 ≤ 642) and (19 ≤ NEM1 ≤ 36)) Then P	0.785
Y	1	if((663 ≤ NH3 ≤748) and (105 ≤ NO2 ≤248) and (784 ≤ T2620 ≤937) and (170 ≤ MQ7 ≤183) and (279 ≤ MQ6 ≤323) and (82 ≤ MQ4 ≤284) and (29 ≤ TEMP1 ≤36)) Then A	0.786
	2	if((254 ≤ MQ8 ≤273) and (119 ≤ NO2 ≤238) and (751 ≤ T2620 ≤932) and (251 ≤ MQ6 ≤332) and (141 ≤ MQ4 ≤277) and (27 ≤ TEMP1 ≤36)) Then A	0.776
	3	if((923 ≤ T2602 ≤955) and (498 ≤ T822 ≤796) and (166 ≤ HCHO ≤270) and (134 ≤ MQ7 ≤250)) Then A	0.765
	4	if((385 ≤ MQ3 ≤549) and (735 ≤ NH3 ≤792) and (910 ≤ T2602 ≤972) and (263 ≤ MQ6 ≤359) and (118 ≤ MQ4 ≤288) and (21 ≤ NEM1 ≤33)) Then A	0.755

**A**: Absence CL, **P**: Presence CL.

The most successful rules obtained have been cross‐checked with the test data. Tables 13 and 14 display a comparison of the results of the proposed method with standard rule‐based and classification methods. The findings in Tables 13 and 14 indicate that the proposed rule‐based Parallel SFOA method is significantly superior to all other considered methods. The statistical findings of ten independent experiments with the rule‐based Parallel SFOA are presented in Table 15. Upon analysing the statistical results, it can be observed that the values of Class A outperform those of Class P.

**TABLE 13:** The comparative results of the rule‐based Parallel SFO and well‐known rule‐based methods (weighted), according to presence CL.

Method	Accuracy
Rule‐based Parallel SFO	79.34
Jrip	74.44
Ridor	73.82
Furia	74.43
OneR	70.14
ConjuctiveRule	74.43
DecisionTable	74.43
ZeroR	74.44

**TABLE 14:** The comparative results of the proposed rule‐based Parallel SFO and standard classification methods (weighted), according to presence CL.

Method	Accuracy
Rule‐based Parallel SFO	79.34
NB	72.39
SVM(SMO)	74.02
KStar	63.59
Vote	74.44
HyperPipes	28.22
J48	74.44
RandomTree	62.78

**TABLE 15:** The statistical accuracy results of all the rule‐based Parallel SFO experiments, according to Presence CL.

	Class P	Class A
Best	80.60	79.60
Worst	43.90	64.30
Mean	71.00	73.00
Median	71.00	74.00
Std	0.06	0.03

According to changes in the uterine gas environment, the occurrence of the CL was at 80.60%, while its absence was at 79.60%. In the same context of uterine gas environment changes, the occurrence of follicles larger than 1.5 cm in the ovaries was determined to be 82%, in contrast to an occurrence rate of 80% for follicles smaller than 1.5 cm.

## 4 Discussion

Achieving maximum accuracy in heat detection is critical for the success of automated systems designed to identify cows ready for insemination. Of the major signs of heat, standing to be mounted and mounting behaviour are deemed as the most significant visible cues for heat detection in cattle (Reith and Hoy 2018; Yoshida and Nakao 2005). Regrettably, a reduction in these behaviours has been primarily seen in high‐yielding cows (At‐Taras and Spahr 2001). A marked reduction in these behaviours has also been noted in paddocks with reduced space per cow and slippery floors. These circumstances restrict the application of programs that rely on the determination of such parameters.

The capacity to accurately recognise estrus using non‐invasive electronic nose technology is reported (Ali et al. 2022). Similar technologies have employed disposable swabs, taken from the perineal region, to detect estrus (Lane and Wathes 1998; Manzoli et al. 2019). It is recognised that estrus in cows is associated with a variety of volatile compounds, including amines, ethers, diols, alcohols and ketones, especially elevated concentrations of methylheptanol and acetaldehyde, some of which might serve as pheromones (Archunan, Rajanarayanan, and Karthikeyan 2014). Body secretions like urine, feces, milk, sweat and vaginal fluid are presumed to have pheromonal functions in cattle, influencing both sexual and social behaviour (Archunan, Rajanarayanan, and Karthikeyan 2014; Sankar and Archunan 2011; Sankar and Archunan 2008; Rivard and Klemm 1989). Despite intensive research, no systems have been identified that can detect estrus based on the changes in uterine environment gases. Metrisör represents the first such device. According to the data from this study, the Metrisör successfully detected the presence of CL at a rate of 80.60% and the absence at a rate of 79.60%, according to the uterine environment's gas alterations. Likewise, the device identified the existence of follicles over 1.5 cm in the ovaries at 82% and those below 1.5 cm at 80%, based on the uterine environment's gas modifications. Metrisör can detect the changes of 25 different gases such as CO_2_, NO_2_, NH_3_, benzene, ethanol, CH_4_, H_2_, CO, NH_4_, toluene, formaldehyde, O_2_, C_4_H_10_, acetone, N‐hexene, methanol, and general VOC with its 17 sensors.

## 5 Conclusion

The project team's study demonstrates that the Metrisör device they developed, which utilises sensor technology, can accurately determine the presence of a CL in the ovary and detect follicles larger or smaller than 1.5 cm. This is achieved by identifying gases released into the environment during microorganism reproduction using the SFOA. Therefore, it is concluded that this new approach can quickly, reliably, and effectively determine the period in an animal's sexual cycle without the need for a laboratory setting. This solution is possible thanks to the Metrisör's sensor technology, which detects gases released from the reproduction of intrauterine microorganisms.

## Author Contributions

**Ali Risvanli**: Conceptualisation; Funding acquisition; Methodology; Investigation; Formal analysis; Visualisation; Writing–original draft. **Burak Tanyeri**: Conceptualisation; Data curation; Software. **Güngör Yildirim**: Resources; Supervision; Validation. **Yetkin Tatar**: Methodology; Investigation. **Mehmet Gedikpinar**: Conceptualisation; Formal analysis; Writing–review & editing. **Hakan Kalender**: Funding acquisition; Resources; Supervision. **Tarık Safak**: Formal analysis**;** Investigation. **Burak Yuksel**: Formal analysis**;** Investigation. **Burcu Karagulle**: Formal analysis**;** Investigation. **Oznur Yilmaz**: Formal analysis**;** Investigation. **Cebrail Barut**: Formal analysis**;** Investigation. **Mehmet Akif Kilinc**: Methodology; Investigation.

## Ethics Statement

The ethics committee approval was obtained from the Fırat University Animal Experiments Local Ethics Committee (FU‐2019/11).

## Conflicts of Interest

The authors declare no conflicts of interest related to this report.

### Peer Review

The peer review history for this article is available at https://www.webofscience.com/api/gateway/wos/peer‐review/10.1002/vms3.70252.

## Data Availability

This publication contains all data generated or analysed during the study, including supplementary information files.
